# Expellency, anti-feeding and speed of kill of a dinotefuran-permethrin-pyriproxyfen spot-on (Vectra®3D) in dogs weekly challenged with adult fleas (*Ctenocephalides felis*) for 1 month—comparison to a spinosad tablet (Comfortis®)

**DOI:** 10.1007/s00436-015-4470-7

**Published:** 2015-04-15

**Authors:** Marie Varloud, Josephus J Fourie, Byron L Blagburn, Audrey Deflandre

**Affiliations:** Ceva Santé Animale S. A, 10 Avenue de la Ballastière, 33500 Libourne, France; ClinVet International (Pty) Ltd, P.O. Box 11186, Universitas, Bloemfontein, 9321 South Africa; College of Veterinary Medicine, Auburn University, Auburn, AL 36849 USA

**Keywords:** Flea, Insecticidal, Anti-feeding, Blood meal, *Canis lupus familiaris*, Control, Prevention

## Abstract

This study was designed to compare the efficacy of two ectoparasiticides against adult fleas on dogs: a topical (DPP, dinotefuran-permethrin-pyriproxyfen) and a systemic (S, spinosad). Dogs (*n* = 48; 10.21–22.86 kg BW) were allocated to six groups of eight dogs each (C1, C4, DPP1, DPP4, S1, S4). Dogs in the treated groups were administered a topical (3.6 mL of DPP) or a tablet (665 or 1040 mg of S) on day 0. Infestations with 100 unfed fleas (*Ctenocephalides felis*) occurred on days −6, −1, 2, 7, 14, 21 and 28. An additional untreated group (QC, *n* = 6) was involved to evaluate the flea-anti-feeding efficacy. These dogs were infested once with 150 fleas prior to combing of at least 50 live fleas from each dog 5 or 10 min after infestation. In the treated group, dislodged dead and moribund fleas were collected from dogs 5, 10, 15 and 60 min (DPP1, S1) or 5, 10, 30 and 240 min (DPP4, S4) post-treatment and subsequent flea infestations on pans placed underneath the cages. Fleas were counted and removed from dogs by combing 1 (C1, DPP1, S1) or 4 h (C4, DPP4, S4) post-treatment and subsequent infestations. Quantitative PCR analysis of the canine cytochrome b gene was conducted on dislodged fleas collected from treated and control (QC) dogs 5 and 10 min after post-treatment infestations. The number of gene copies was used as a marker of blood volume ingested by fleas. Dislodgeability and insecticidal efficacy were calculated using arithmetic means. A rapid onset of killing was observed for DPP with 12.7 % of dead and moribund fleas being dislodged in average from dogs as soon as 5 min after infestation. DPP exhibited a significantly higher and sustained speed of kill than S. The average insecticidal efficacy was 86 ± 8.8 and 95.3 ± 2.1 % with DPP, whereas it was only 33.7 ± 19.9 and 57.6 ± 18.6 % with S at respectively 1 and 4 h after weekly reinfestations. The DPP combination significantly inhibited the feeding of fleas (89 % reduction) up to onset of flea mortality for 1-month post-treatment.

## Introduction

Dogs are often exposed to flea infestations that can be severe. The cat flea (*Ctenocephalides felis felis*) is the most frequent species encountered on dogs. Adult females and males are structurally specialized to colonize their host’s coat. They acquire repeated blood meals by probing the skin with their mouthparts. The kinetics of blood feeding was investigated on cats for *C. felis* (Cadiergues et al. 2000) and on dogs for *C. canis* (Cadiergues et al. 2001) demonstrating that blood can be detected within 5 min after infestation. A recent molecular study indicated that *C. felis* fleas began feeding on dog blood in the same time interval (Wang et al. 2010). Dogs can become hypersensitive to flea bites (Lee et al. 1997) and may develop clinical dermatitis (Bruet et al. 2012). Flea feeding also provides the opportunity for transmission of pathogens such as *Rickettsia* spp. Once fleas are fed, blood digestion occurs and about 30 min after infestation (Wang et al. 2012), they start producing faeces containing host blood, also known as flea dirt. These excreta can be infectious and are considered as the route of transmission of *Bartonella* (Bouhsira et al. 2013; Kernif et al. 2014) or viruses (Mencke et al. 2009). Ectoparasiticides are expected not only to eliminate fleas but also to prevent further infestations as well as the associated risk of disease transmission. To achieve this objective, products must exhibit a rapid onset of action and sustained levels of efficacy until the next administration. This experiment was designed to investigate the insecticidal and anti-feeding efficacy of a topical ectoparasiticide (Vectra®3D, DPP) in dogs weekly challenged by *C. felis* fleas for 1 month. A systemic insecticide (Comfortis®, S) was used to compare flea dislodgeability and adulticidal efficacy.

## Materials and methods

The animals were not treated by any individual involved in performing the post-treatment assessments and observations. Study groups were coded to blind the assessors.

### Dogs

Forty-eight healthy mongrel dogs (>6 months old, 1:1 sex ratio) were enrolled for the study and started an acclimation period of at least 7 days. Because removal and sampling of fleas was required through combing at 5- and 10-min post-infestation from untreated animals, an additional untreated group (*n* = 6, CQ) was specifically enrolled in parallel to quantify flea feeding. The dogs had not been treated with an acaricide or insecticide for 16 weeks. All dogs were identified with electronic transponders and were dewormed at the beginning of the study. Their body weight (BW) ranged from 10.21 to 22.86 kg, and their hair length ranged from 11 to 39 mm. The dogs were housed individually in an indoor animal unit, controlled for temperature with 12 h light:12 h darkness photoperiod. Humidity was recorded daily with a hygrometer. Dogs were fed commercial dog food once daily with water available ad libitum. No contact between dogs was possible during the study. All dogs were observed for general health status and adverse reactions to treatment once daily, from day −7 to day 28, except on day 0, where specific health observations were made hourly for 4 h after treatment. On day −7, 1 mL blood was collected in EDTA tubes from each animal prior to white blood cell counts. This protocol was approved by an independent animal ethics committee.

### Allocation

The study followed a randomized block design. The 48 dogs were ranked, within gender, in descending order of individual day −5 flea counts. Within each gender, animals were blocked into blocks of 8 dogs each. Within each block, dogs were randomly allocated to one of the six groups.

### Treatment

Each dog was treated with the allocated treatment on day 0. Dogs in groups C1 and C4 were untreated. Dogs in groups DPP1 and DPP4 were treated with the commercial formulation DPP containing the active ingredients dinotefuran (4.95 %, *w*/*w*), pyriproxifen (0.44 %, *w*/*w*) and permethrin (36.08 %, *w*/*w*). Dogs in groups S1 and S4 were treated with the commercial formulation S containing the active ingredients spinosad. DPP was administered topically, as a spot-on, according to the manufacturer’s label directions, at a rate of 3.6 mL per dog applied equally (1.2 mL per site) in three spots at the shoulder blades, the mid-back and the base of tail. S was administered orally: one 665 mg tablet was administered to the animals weighing 9.5 to 14.7 kg while one 1040 mg tablet was administered to the animals weighing 14.8 to 23.1 kg. Dogs were given the possibility to take the pill spontaneously. If the pill was not taken spontaneously, the dogs were force-fed. Dogs were fed immediately after dosing with S.

### Flea infestations

A laboratory-bred strain of *C. felis felis* (European origin), routinely fed on cats, was used in the experimental infestation. The fleas were unfed and of mixed sex. Each dog was infested with 100 adult fleas on days −6, −2, 2, 7, 14, 21 and 28, except the CQ-untreated group which was infested once with 150 fleas prior to combing of at least 50 live fleas from each dog 5 and 10 min after infestation.

In the treated groups, dislodged dead and moribund fleas were collected at 5, 10, 15 and 60 min after flea infestations for DPP1 and S1 and at 5, 10, 30 and 240 min after flea infestations for DPP4 and S4. These fleas were collected on pans placed underneath the cages on days 2, 7, 14, 21 and 28. In all groups, fleas were counted and removed from dogs by combing, respectively, 1 h ± 5 min or 4 h ± 5 min post-treatment and subsequent infestations (Table 1). A fine-toothed flea comb was used to recover fleas from the animal’s fur. Several strokes of the comb were applied in each body area. This procedure was performed at least twice and until no more live fleas were found. At each assessment, fleas were classified as live, moribund or dead.

**Table 1:** Study design and time interval between flea infestations and assessments

Effect measured	Groups of dogs						
	C1 (*n* = 8)	C4 (*n* = 8)	DPP1 (*n* = 8)	DPP4 (*n* = 8)	S1 (*n* = 8)	S4 (*n* = 8)	CQ (*n* = 6)
Dislodgeability	Not relevant		5 min 10 min	5 min 10 min	5 min 10 min	5 min 10 min	Not relevant
Anti-feeding			5 min 10 min	5 min 10 min	5 min 10 min	5 min 10 min	5 min 10 min
Insecticidal	1 h	4 h	1 h	4 h	1 h	4 h	–

*C* control untreated; *DPP* dinotefuran, pyriproxyfen, permethrin; *S* spinosad; *CQ* control untreated for quantification of blood intake

### Quantification of flea feeding

Real-time quantitative PCR analysis of the canine cytochrome b gene (Woods et al. 2009) was conducted on pools of ten randomly selected dislodged fleas collected from individual treated dogs at 5 (*n* = 16) and 10 min (*n* = 16) after each weekly infestation (Table 1). For optimal reliability, quantitative assessments must be performed shortly after infestation, before blood saturation of fleas and the excretion of bloody faeces which is expected to occur approximately 30 min after infestation (Wang et al. 2012). The analysis was also performed on pools of ten randomly selected live fleas combed from individual control (CQ) dogs 5 (*n* = 3) or 10 min (*n* = 3) after infestation. The number of canine gene copies was used as a marker of blood volume ingested by fleas. Fleas were preserved in sterile tubes containing 70 % ethanol before being washed in phosphate buffer saline prior to DNA isolation (Han et al. 2006).

### Statistical analysis

The cumulative arithmetic means (CAM) of dead and moribund fleas dislodged from dogs were calculated for each treatment 5 and 10 min after infestations. The groups were compared by an ANOVA with a treatment effect. Statistical significance was declared at a two-sided *p* value of 0.05.

The geometric means (GM) of canine cytochrome b gene copies in fleas dislodged from dogs were calculated for DPP, 5 and 10 min after infestations. GM calculations were based on the means of the average canine cytochrome b gene copies in fleas (average + 1) data. One (1) was subsequently subtracted from the result to obtain a meaningful value for the GM of the study groups. Percent of feeding inhibition for each assessment time (5 min or 10 min) on each day was calculated as:

$$ \mathrm{Feeding}\ \mathrm{inhibition}\kern0.5em \left(\%\right)=100\times \frac{\left(\mathrm{MCcytb}-\mathrm{MTcytb}\ \right)}{\mathrm{MCcytb}} $$

where:

MC_cytb_: Mean (GM or AM) of the average canine cytochrome b gene per fleas on dogs in the C1 and C4 groups and
MT_cytb_: Mean (GM or AM) of the average canine cytochrome b gene per fleas on dogs in the DPP1 and DPP4 groups.

The AM and GM were calculated for live fleas collected from dogs in the respective groups 1 and 4 h post-treatment and subsequent infestations. Moribund fleas were considered as a failure and counted as live ones. Calculations of GM were based on the means of the live and moribund flea (count + 1) data. One (1) was subsequently subtracted from the result to obtain a meaningful value for the GM of the study groups. The groups were compared using a one-way ANOVA with a treatment effect after logarithmic transformation on the (flea count +1) data. Unadjusted pair-wise comparisons were performed between each pair of groups with ANOVA contrasts. Statistical significance was declared at a two-sided *p* value of 0.05. Groups were also compared by a non-parametric analysis using the Kruskal-Wallis test. Unadjusted pair-wise comparisons were performed between each pair of groups with the Mann-Whitney test. Percent of insecticidal efficacy for each assessment time (1 h or 4 h) on each day was calculated as:

$$ \mathrm{Efficacy}\kern0.5em \left(\%\right)=100\times \frac{\left(\mathrm{M}\mathrm{C}-\mathrm{M}\mathrm{T}\ \right)}{\mathrm{MC}} $$

where:

MC: Mean (GM or AM) of live and moribund fleas in the control groups and
MT: Mean (GM or AM) of live and moribund fleas in the respective treated groups.

### Guidelines

The study was conducted according to the Good Clinical Practices (CVMP 2000). The study was conducted in accordance with the current and appropriate guidelines (CVMP 2007).

## Results

The study was run in November and December which corresponds to summer in South Africa. The temperature inside the housing unit remained between 16.8 and 24.0 °C, and the recorded relative humidity ranged from 18.5 to 82.9 %.

The BW and hair length of dogs were homogenous between groups (Table 2). The pre-treatment white blood cell counts were within the normal reference range for dogs. The DPP treatment delivered 12.1 ± 3.0 mg/kg BW of dinotefuran, 88.2 ± 21.9 mg/kg BW of permethrin and 1.1 ± 0.3 mg/kg BW of pyriproxyfen. The S treatment delivered 54.8 ± 7.1 mg/kg BW of spinosad (Table 3). Four out of 16 of the dogs given S took the pill spontaneously. Seventy-five percent of the dogs treated with S were force-fed.

**Table 2:** Body weights and average hair length of dogs on day −4

	Dogs body weight (kg)			Dogs hair length (mm)		
Group	Mean	Min	Max	Mean	Min	Max
C1	16.44	12.70	22.86	21.66	15.75	29.75
C4	17.14	10.90	22.66	22.75	11.25	33.00
DPP1	16.31	10.93	21.89	21.72	15.25	31.25
DPP4	17.89	12.87	21.92	20.81	16.50	24.50
S1	18.39	13.55	22.25	22.53	14.75	38.75
S4	15.68	10.21	20.65	20.69	14.00	27.25

*C* control untreated; *DPP* dinotefuran, pyriproxyfen, permethrin; *S* spinosad

**Table 3:** Concentrations of actives delivered individually in each group on day 0

Groups	Actives	mg/kg BW			mg/m^2^ BSA		
		Mean	Min	Max	Mean	Min	Max
DPP1	Dinotefuran	12.7	9.0	17.9	301.2	239.9	382.0
	Pyriproxyfen	1.1	0.8	1.6	26.8	21.3	34.0
	Permethrin	92.5	65.3	130.7	2195.2	1748.5	2784.6
DPP4	Dinotefuran	11.5	8.9	15.2	282.3	239.7	342.4
	Pyriproxyfen	1.0	0.8	1.4	25.1	21.3	30.4
	Permethrin	83.9	65.2	111.0	2057.7	1746.9	2495.9
S1	Spinosad	51.6	46.2	61.2	Not relevant		
S4	Spinosad	57.9	48.2	68.0			

*BW* body weight, *BSA* body surface area

No abnormal signs were observed in any of the treated dogs during the 4-h post-treatment.

**Fig. 1:**
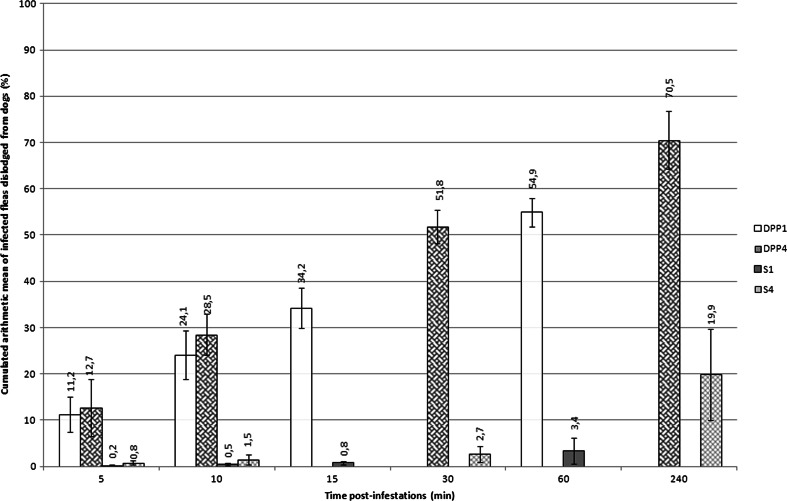
Kinetic of cumulative arithmetic means of dead and moribund fleas dislodged after artificial infestation with 100 adult unfed fleas (*Ctenocephalides felis*) in four groups of 8 dogs treated with dinotefuran-permethrin-pyriproxyfen (DPP) or spinosad (S)

On dogs with an existing flea infestation, the CAM number of dislodged fleas remained under 3 % until 1-h post-treatment regardless of the treatment (Tables 4 and 5). Four hours after treatment, the CAM number of dislodged fleas found dead or moribund was higher (*p* < 0.05) in the DPP-treated dogs (58.6 %) as compared to the S-treated dogs (24.1 %). On the DPP-treated dogs, the average CAM number of dislodged fleas increased gradually between 5 min (11.2 ± 5.7 % in DPP1 and 12.7 ± 7.5 % in DPP4) and 4 h (70.5 ± 7.4 % in DPP4) after each weekly infestation (Fig 1). The CAM number of dislodged fleas was higher (*p* < 0.05) in the DPP-treated dogs as compared to S-treated dogs at every time-point, except 5 min after infestation on days 2, 7 and 21 for DPP1 and S1 and on day 7 for DPP4 and S4 (Tables 4 and 5).

**Table 4:** Cumulative arithmetic mean flea counts of parasites dislodged (dead or moribund) from dogs 5, 10, 15 and 60 min after weekly infestations (days 2, 7, 14, 21 and 28) with adult *Ctenocephalides felis* on dogs treated with a topical (DPP, *n* = 8) or an oral (S, *n* = 8) ectoparasiticide on day 0

	Time post-infestation (min)							
	5		10		15		60	
Days	DPP1	S1	DPP1	S1	DPP1	S1	DPP1	S1
0	0.0	0.4	0.6	0.5	0.8	0.5	2.9	1.3
2	9.1	0.5	16.8	0.8^a^	27.6	1.3^a^	58.0	7.8^a^
7	10.1	0.3	21.8	0.6^a^	33.4	0.6^a^	50.8	4.1^a^
14	14.6	0.1^a^	30.5	0.6^a^	38.5	0.6^a^	55.4	2.6^a^
21	6.5	0.1	23.6	0.5^a^	33.8	1.0^a^	52.9	1.4^a^
28	15.5	0.1^a^	27.8	0.1^a^	37.9	0.5^a^	57.6	0.9^a^
Mean (%)	11.2	0.2	24.1	0.5	34.2	0.8	54.9	3.4
sd	5.7	0.2	10.7	0.2	14.2	0.3	21.4	2.6

*DPP* dinotefuran, pyriproxyfen, permethrin; *S* spinosad

^a^Treated groups differed from each other (*p* < 0.05) within each time-point

**Table 5:** Cumulative arithmetic mean flea counts of parasites dislodged (dead or moribund) from dogs 5, 10, 15 and 60 min after weekly infestations (days 2, 7, 14, 21 and 28) with adult *Ctenocephalides felis* on dogs treated with a topical (DPP, *n* = 8) or an oral (S, *n* = 8) ectoparasiticide on day 0

	Time post-infestation (min)							
	5		10		30		240	
Days	DPP4	S4	DPP4	S4	DPP4	S4	DPP4	S4
0	0	0.5	0	0.5	1.0	0.9	58.6	24.1^a^
2	22.9	0.6^a^	33.0	1.4^a^	56.5	4.0^a^	81.4	31.3^a^
7	9.9	1.0	25.8	2.0^a^	49.0	4.1^a^	68.0	25.3^a^
14	11.6	1.4^a^	33.6	3.0^a^	53.4	3.8^a^	68.6	21.5^a^
21	6.6	0.5^a^	24.4	0.9^a^	47.5	1.3^a^	65.8	16.4^a^
28	12.5	0.3^a^	25.8	0.3^a^	52.8	0.5^a^	68.5	5.3^a^
Mean (%)	12.7	0.8	28.5	1.5	51.8	2.7	70.5	19.9
sd	7.5	0.4	12.3	1.0	21.0	1.7	7.4	9.0

*DPP* dinotefuran, pyriproxyfen, permethrin; *S* spinosad

^a^Treated groups differed from each other (*p* < 0.05) within each time-point

**Fig. 2:**
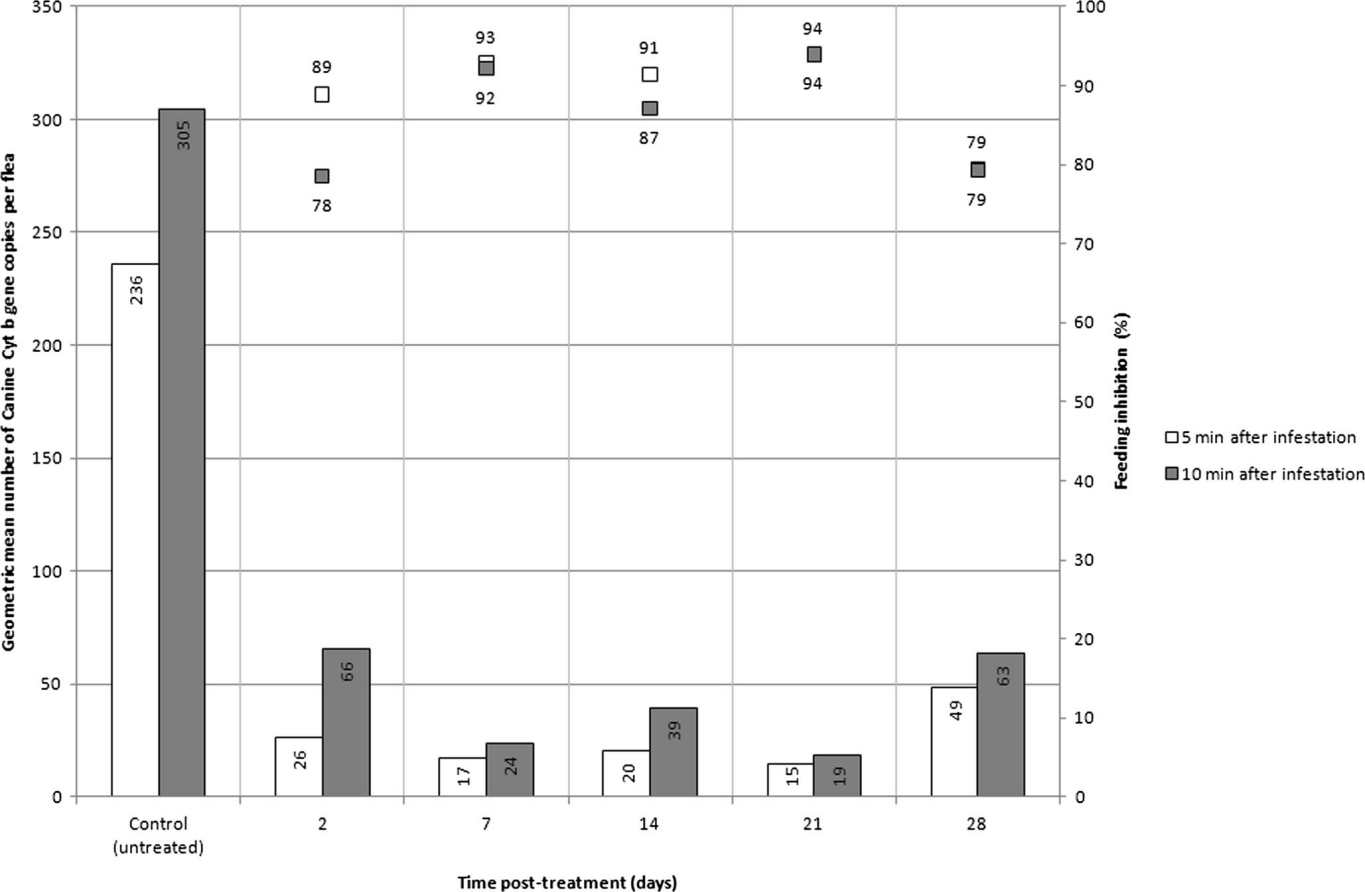
Weekly assessment over 1 month of the geometric mean number of canine cytochrome b gene copies in fleas dislodged from dogs treated with dinotefuran-permethrin-pyriproxyfen (DPP) on day 0 and compared to fleas removed from untreated dogs 5 or 10 min after infestation

In CQ dogs, the GM number of canine cytochrome b gene copies per flea was 235.9 and 304.6 as measured from fleas combed 5 or 10 min after infestation, respectively (Table 6). In the DPP-treated dogs, the GM number of cytochrome b gene copies per flea ranged between weekly challenges from 6.4 to 55.5 in fleas collected 5 min after infestation and from 11.4 to 75.9 in fleas collected 10 min after infestation (Fig. 2). The average feeding inhibition was 89.3 and 86.2 % when measured 5 and 10 min after infestation of the DPP-treated dogs, respectively. In the S-treated dogs only 0.8 ± 0.4 and 1.5 ± 1.0 fleas were dislodged in average 5 and 10 min after reinfestations. It was therefore not possible to perform a reliable assessment of the quantity of blood taken by fleas from these dogs.

**Table 6:** Geometric mean number of cytochrome b gene copies per flea from dogs 5 and 10 min after weekly infestations (days 2, 7, 14, 21 and 28) on 16 dogs treated with a topical (DPP) or after a single infestation on untreated dogs (CQ)

	Time post-infestation (min)									
	5	10	5				10			
Days	CQ	CQ	DPP1 (*n* = 8)	DPP4 (*n* = 8)	DPP mean	FI (%)	DPP1 (*n* = 8)	DPP4 (*n* = 8)	DPP mean	FI (%)
2	235.9	304.6	10.6	43.3	26.2	88.9	57.5	75.9	65.5	78.5
7			43.0	6.4	17.1	92.8	21.7	26.2	23.9	92.2
14			21.4	19.5	20.4	91.3	50.3	31.8	39.4	87.0
21			15.5	14.1	14.6	93.8	29.7	11.4	18.5	93.9
28			55.5	42.3	48.5	79.5	63.8	62.8	63.3	79.2
Mean			29.2	25.2	25.4	89.3	44.6	41.6	42.1	86.2
sd			19.2	16.8	13.6	5.8	18.1	26.8	21.8	7.1

*CQ* control untreated for quantification of blood intake; *DPP* dinotefuran, pyriproxyfen, permethrin; *FI* feeding inhibition (%)

In the C1 and C4 groups, the average flea combing recovery rate was 80.4 ± 14.1 % (Tables 7 and 8). On treated dogs with an existing flea infestation, the AM flea counts 1 h after treatment were above 60. In the DPP group, the AM flea count was 61.4, of which 5.6 fleas in average were moribund. In the S group, the AM flea count was 64.1, of which 0.6 fleas in average were moribund. Four hours after treatment the flea counts were lower (*p* < 0.05) in the treated groups (DPP or S) than in the control group (C). None of the treated dogs was free of fleas when assessed 1 h after treatment. Four hours after treatment, only four of the DPP-treated dogs were free of fleas (Table 9). On the DPP-treated dogs, the flea counts 1 and 4 h after infestations were lower (*p* < 0.05) than in the control group for every weekly infestation. The flea counts from the S-treated dogs were lower (*p* < 0.05) than in the control group on days 2, 7 and 14 when assessed 1 h post-infestation and on days 2, 7, 14 and 21 when assessed 4 h post-infestation. The flea counts observed 1 and 4 h after infestation from the DPP-treated dogs were lower (*p* < 0.05) than from the S-treated dogs at every infestation. Insecticidal efficacy 1 h after infestations ranged between 73 and 95 % AM on DPP-treated dogs whereas it ranged from 12 to 50 % on S-treated dogs. Except on day 28 (92 %), efficacy was above 95 % AM 4 h after weekly infestation of DPP-treated dogs. Efficacy ranged from 31 to 75 % AM 4 h after weekly infestation of S-treated dogs. None of the S-treated dogs was free of fleas when assessed 1 or 4 h after infestations, except 4 h after infestations on days 2 (4/8) and 7 (2/8). Among the DPP-treated dogs, 3 to 5 were free of fleas 1 h after infestation on day 7, 14 and 21. Flea-free dogs were observed 4 h after each infestation. On day 2, all the DPP-treated dogs were free of fleas 4 h after infestation.

**Table 7:** Flea counts 1 h after treatment and weekly infestations (days −2, 2, 7, 14, 21 and 28) with adult *Ctenocephalides felis* on dogs untreated (C) or treated with a topical (DPP) or an oral (S) ectoparasiticide on day 0

	C1		DPP1		S1	
Days	AM	GM	AM	GM	AM	GM
0	72.5	71.8	61.4	59.3	64.1	62.8
2	95.3	95.0	26.1	17.7^a^	51.5	49.5^a, b^
7	88.4	87.1	9.0	5.5^a^	44.5	41.0^a, b^
14	87.8	87.0	4.5	2.4^a^	44.8	42.4^a, b^
21	81.8	81.5	7.9	2.7^a^	72.0	70.9^b^
28	79.8	79.4	14.1	11.4^a^	70.1	69.0^b^

*C* control untreated; *DPP* dinotefuran, pyriproxyfen, permethrin; *S* spinosad; *AM* arithmetic mean; *GM* geometric mean

^a^Treated groups (DPP or S) differed from control group (C) (*p* < 0.05) within each time-point

^b^Treated groups differed from each other (*p* < 0.05) within each time-point

**Table 8:** Flea counts 4 h after treatment and weekly infestations (days −2, 2, 7, 14, 21 and 28) with adult *Ctenocephalides felis* on dogs untreated (C) or treated with a topical (DPP) or an oral (S) ectoparasiticide on day 0

	C4		DPP4		S4	
Days	AM	GM	AM	GM	AM	GM
0	70.3	69.3	10.5	6.0^a^	28.6	16.3^a^
2	80.9	80.1	2.9	0.8^a^	22.3	12.^a, b^
7	77.3	75.8	3.6	1.8^a^	19.0	11.5^a, b^
14	73.4	71.2	1.9	1.1^a^	27.4	17.1^a, b^
21	81.5	80.2	3.6	2.1^a^	43.8	33.1^a, b^
28	76.5	75.0	6.3	3.4^a^	52.6	49.9^b^

*C* control untreated; *DPP* dinotefuran, pyriproxyfen, permethrin; *S* spinosad; *AM* arithmetic mean; *GM* geometric mean

^a^Treated groups (DPP or S) differed from control group (C) (*p* < 0.05) within each time-point

^b^Treated groups differed from each other (*p* < 0.05) within each time-point

**Table 9:** Efficacy 1 and 4 h after treatment and weekly infestations (days −2, 2, 7, 14, 21 and 28) with adult *Ctenocephalides felis* on dogs untreated (C) or treated with a topical (DPP) or an oral (S) ectoparasiticide on day 0

	DPP 1 h			DPP 4 h			S 1 h			S 4 h		
Days	AM	GM	FF	AM	GM	FF	AM	GM	FF	AM	GM	FF
0	15.3	17.5	0/8	85.1	91.3	4/8	11.6	12.6	0/8	59.3	76.6	0/8
2	72.6	81.4	0/8	96.4	99.1	8/8	45.9	47.9	0/8	72.5	84.1	4/8
7	89.8	93.7	3/8	95.3	97.7	5/8	49.6	52.9	0/8	75.4	84.8	2/8
14	94.9	97.2	5/8	97.4	98.4	6/8	49.0	51.3	0/8	62.7	76.0	0/8
21	90.4	96.6	4/8	95.6	97.4	4/8	11.9	12.9	0/8	46.3	58.7	0/8
28	82.3	85.6	0/8	91.8	95.5	3/8	12.1	13.1	0/8	31.2	33.5	0/8

*C* control untreated; *DPP* dinotefuran, pyriproxyfen, permethrin; *S* spinosad; *AM* efficacy calculated on arithmetic mean; *GM* efficacy calculated on geometric mean; *FF* number of dogs free of live fleas

## Discussion

As demonstrated in the C group with over 80 % of average flea combing recovery rate, the flea challenges were vigorous during the whole duration of the study. Fleas collected 5 or 10 min after infestation from the CQ-untreated dogs exhibited large numbers of canine cytochrome b gene copies per flea which confirmed early blood feeding behaviour of these parasites as measured with a similar method but using a different canine gene target (Wang et al. 2010, 2012). Indeed, about 21 % of *C. canis* fleas had begun a blood meal within 5 min on dogs (Cadiergues et al. 2001) while about 25 % of *C. felis* fleas were engorged from cats in this time interval (Cadiergues et al. 2000). The results from the present study confirmed that at least 1 out of 10 *C. felis* fleas can start to feed on dog blood within 5 min after infestation.

Therapeutic and residual performances of the DPP and S treatments were evaluated through flea dislodgeability and insecticidal efficacy. On already treated dogs, flea feeding inhibition was investigated from fleas spontaneously dislodged from DPP-treated dogs.

### Therapeutic efficacy

On already infested dogs, the number of dislodged fleas increased dramatically between 1 and 4 h after treatment. At this last time-point, there was more than twice more fleas being dislodged dead or moribund from the DPP-treated dogs (58.6 %) as compared to the S-treated dogs (24.1 %). Some moribund fleas did not fall from the dogs (5.6 AM at 1 h and 3.6 AM at 4 h for DPP) and were considered as a failure. The DPP treatment exhibited indeed a flea insecticidal AM efficacy of 15.3 % in 1 h and 85.1 % in 4 h. These results are in agreement with previous assessment of DPP performances against fleas in dogs that showed above 95 % insecticidal efficacy in 6 h after treatment (Dryden et al. 2011; Varloud et al. 2014). On the contrary, the S treatment exhibited an insecticidal AM efficacy of only 11.6 % in 1 h and 59.3 % in 4 h. These results are in contradiction with the 100 % efficacy expectations for S in 4 h of treatment (Blagburn et al. 2010). However, AM efficacy of this product was already reported at 81 % when measured on dogs 4 h after treatment against *C. canis* (Franc and Bouhsira 2009). These results confirmed that a topical parasiticide (DPP) applied as a spot-on can provide a faster onset of action against fleas than a systemic one (S) delivered orally to infested dogs (Dryden et al. 2011).

### Residual efficacy

On already treated dogs, DPP quickly started (5 min) expelling fleas from treated dogs and dislodged in 4 h more than 65 % of fleas during 1 month after treatment. Because the DPP product is applied on the skin and spreads via the skin and haircoat of the animals (CVMP 2013), it comes immediately in contact with the parasite where the infestation occurs. Permethrin alone is known for its knock-down effect against *C. felis* fleas from dogs. Seven days after treatment with a permethrin spray (2 %, 5 mL/kg), 97 and 100 % of fleas were dislodged from dogs in 1 and 2 h, respectively (Ascher et al. 1998). No live fleas were found from dogs treated with a dinotefuran (22 %, *w*/*w*)–pyriproxyfen (3 %, w/w) combination only 6 h after treatment or infestation until day 28 (Dryden et al. 2011). This study demonstrates that, when combined together in a single product, permethrin and dinotefuran can dislodge a considerable proportion of fleas from dogs for 1 month after administration. This effect contributes greatly to the speed of kill performances of DPP. On contrary, fleas did not start falling from the S-treated dog before 4 h post-infestation. On day 28, the average proportion of fleas expelled 4 h after infestation from these dogs was 5.3 %. This is less than the 6.5 % of fleas that were recorded to fall dead from untreated dogs 32 h after infestation (Mahoney et al. 2001).

The flea insecticidal efficacy measured 1 h after infestations of the DPP-treated dogs was above 80 % AM and 85 % GM from day 7 to day 28. When measured 4 h after infestation, it was above 90 % AM and 95 % GM from day 2 until day 28 after administration. These results confirmed previous observations of the high residual speed of kill of DPP measured 2 (Varloud et al. 2014) and 6 h (Dryden et al. 2011) after infestations. On contrary, when measured on the S-treated dogs, flea insecticidal efficacy was below 85 % AM or GM at every time-point. These performances levels were in agreement with previous observations. The efficacy of S started to decrease between day 7 and 14, as observed against *C. canis* fleas (Franc and Bouhsira. 2009). On day 28, efficacy assessed 4 h after infestation was 31 % AM in the present study against *C. felis* fleas and 42 % AM in a previous experiment against *C. canis* fleas (Franc and Bouhsira. 2009). When assessed against the *C. felis* flea strain (KS1), noted for difficulty in achieving and maintaining efficacy with several products, the efficacy of S measured 6 h after infestation was observed to drop to 0 % on day 28 (Dryden et al. 2011).

Through the evaluation of blood uptake from fleas dislodged 5 and 10 min after infestation, this study demonstrated that in average 89 and 86 %, respectively, of flea feeding was inhibited for 1 month after DPP administration. Although a different canine gene marker was used, this is close to the flea feeding inhibition by DPP assessed at 75 and 86 % in live fleas removed from dogs 5 and 10 min after infestation, respectively (Wang et al. 2010). Among other active ingredients and formulations such as imidacloprid, fipronil and dichlorvos/fenitrothion, only a permethrin foam was shown to inhibit over 90 % of flea blood feeding 1 h after treatment or subsequent infestations. However, this effect did not last over 7 days after treatment (Franc and Cadiergues 1998). In DPP, permethrin is combined with dinotefuran in a topical formulation and provides an effective and continuing protection against flea blood feeding in dogs for 1 month after administration. Such an effect could likely affect the transmission of pathogens through blood feeding and through the inhibition of the excretion of potentially infectious faeces by the adult fleas. Inhibition of feeding is also expected to reduce the severity of allergic dermatitis associated with flea feeding.

## Conclusions

This study demonstrated that DPP starts to expel dead or moribund fleas from infested dogs between 1 and 4 h after treatment and that the therapeutic insecticidal efficacy of DPP was higher than S. In situations of prevention against new infestations, over 65 % of fleas were dislodged as dead or moribund from DPP-treated dogs within 4 h after infestation. DPP exhibited higher residual speed of kill than S. It was shown that blood feeding of fleas was quickly (5 min) disrupted by DPP which can therefore be considered as a reliable product strategy against flea infestations in dogs. Further research should be conducted to investigate the viability of the moribund expelled fleas as well as the benefits of DPP in the prevention of flea-borne diseases.
